# Evaluation of the adverse events on applying a functional protocol in patients in an ICU

**DOI:** 10.1186/cc14678

**Published:** 2015-09-28

**Authors:** Maíra J Maturana, Ana Paula O Rodrigues, Gabriela DA Martinelli, Layla H Amarantes, Luiz Alberto M Knaut, Paula TG Serra, Esperidião E Aquim

**Affiliations:** 1 1-Faculdade Inspirar, Curitiba-Pr, São Francisco, Curitiba, Paraná, Brazil

## Introduction

ICU patients are exposed to adverse events, which are defined as unintended complications but are preventable.

## Objective

To identify the adverse effects on the application of the Prófisio Functional Physical Therapy Protocol in critical patients.

## Methods

Experimental study, longitudinal and contemporary, taking place between January and October 2014 with patients admitted to the ICUs of the Trabalhador, Vita Curitiba, Vita Batel, Marcelino Champagnat Hospital and the Neurology Institute of Cutitiba (INC) in the city of Curitiba, PR. The sample was composed of 375 patients, being 57% male and 42.6% female, with an age average of 58 ± 20.9, medium Glasgow and Ramsey 5. The Prófisio Functional Physical Therapy Protocol (Table 1) was applied once a day to patients who were age 18 or older, hemodynamically stable with PAM between 60 and 110 mmHg, whose responsible agreed to sign the TCLE. The hemodynamic variables (heart rate, blood pressure, breathing rate and oxygen saturation) were evaluated before and after the application of the protocol. The adverse effects were defined as loss of central or peripheral venous access, electrodes for cardiac monitoring, intracranial pressure monitoring, external ventricular derivation, removal of urinary catheter, removal of gavage, orthotracheal or tracheostomy tubes, surgical drains, bleedings, and decrease and opening of sutures, and were observed during all application of the protocol.

**Table 1:** The Prófisio Functional Physical Therapy Protocol

	Level 1	Level 2	Level 3	Level 4	Level 5
**A. COGNITIVE CHANGES** **Glasgow<13**	Electrostimulation; Passive Kinesiotherapy.	Postural control; Passive bicycle; Orthostatic board; Outside the ICU ride.	Assisted orthostatism	not applicable	not applicable
**B. COGNITIVE PRESERVED** **Glasgow≥13**	Prevention of deep vein thrombosis (PTVP); Active-assisted kinesiotherapy; Game therapy.	Postural control; Active bicycle; Orthostatic board; Outside the ICU ride.	Standing position, Kinesiotherapy Weathered; Transfer of weight training; March Stationary.	Training march with aid; Squat.	Step; Trampoline; March without assistance; One-leg support.
**Note**	check specific contraindications.	At each level adding exercise described in the table			

*Level 1 *bedridden/restricted to bed due to medical orders, *Level 2 *transfer bed-chair passively, *Level 3 *transfer-bed chair with partial weight, *Level 4 *rambles with partial weight, *Level 5 *wandering without assistance

## Results

A total of 1144 interventions were observed, where only seven (0.61%) showed adverse events. Of the seven only adverse effects, three were classified as light-loss of electrodes of cardiac monitoring-and four were classified as moderate-the unscheduled removal of the gavage, hypotension, drop and loss of surgical drain. The hemodynamic variables did not suffer significant alterations.

## Conclusion

The application of the Prófisio Functional Physical Therapy Protocol showed itself to be safe and with a low risk of adverse effects, when applied to critical patients.
